# Royal Society Conversazione

**Published:** 1924-06

**Authors:** 


### ROYAL SOCIETY CONVERSAZIONE.

Sir Charles Sherrington, G.B.E., and the members of the Royal Society entertained a large company at the conversazione given in the Society's beautiful rooms in Burlington House, on Wednesday, May 14th. As always, there were many interesting exhibits. In the first room cultures of fungi causing dry rot in buildings were shown. Some of the fungi, for example, *Coniophora cerebella*, can only attack wood that is thoroughly damp, as they cannot manufacture water sufficient for their growth. Others, the *Merulius lacrymans*, for instance, when once established, can attack the driest wood, as they render it moist by means of the water they produce and excrete. All the fungi exhibited at Burlington House grow inside timber, but only the two mentioned here can grow over or through other materials such as brick walls, thus causing the rapid destruction of the woodwork of a building. In the next room copies were shown of the original telescopes made by Galileo about 1610, now preserved in the Museo di Fisica at Florence. The longer telescope gives a linear magnification of 14 with a field of 15 minutes of arc, and a resolving power of about 17 seconds of arc.

### Bone-Growth in Pigs.

Keen interest was aroused in the exhibition by the Forestry Commission of eggs of the Green Oak Tortrix Moth (*Tortrix viridana L.*), which causes periodic defoliation in our oak woods. The eggs of this moth are laid in pairs concealed under lichen and bark debris. So far as is known, they have not been found before in England. The eggs were shown as they were found in Surrey on three-year-old oak twigs. Surgeons found the madder-stained preparations illustrating the process of bone-growth in young pigs especially engrossing. These were among the anatomical specimens from the Museum of the Royal College of Surgeons. Madder, it was explained, when taken into the system, stains bone in the act of formation red, but has no effect on bone already formed. It is thus possible by feeding young animals on madder root to observe the progress of the addition of new bone and absorption of the old by which the various parts of the skeleton increase in size and are modelled to their final shape.

### Portrait of Priestley.

A recently discovered portrait in wax of Joseph Priestley, dated 1788, by Percy, a well-known modeller in wax, attracted much attention. It shows Priestley in his robes as a Unitarian minister, with a white stock and grey full-bottomed wig. Next to this portrait was the original silver pocket sun-dial of the Earl of Orrery, made by J. Rowley about 1710. Rowley was the leading mathematical instrument maker of his time, and a contemporary of Sir Christopher Wren, for whom he made the large sun-dial by which the clock of St. Paul's Cathedral, erected by Langley Bradley in 1708, was regulated. During the evening two lectures were given in the meeting room on the ground floor, the first by Mr. F. E. Smith, F.R.S., on " Modern Navigational Devices," and the second by Mr. R. Campbell Thompson on " Travel and Archæology in Mesopotamia."

